# Graph neural network and multi-data heterogeneous networks for microbe-disease prediction

**DOI:** 10.3389/fmicb.2022.1077111

**Published:** 2022-12-22

**Authors:** Houwu Gong, Xiong You, Min Jin, Yajie Meng, Hanxue Zhang, Shuaishuai Yang, Junlin Xu

**Affiliations:** ^1^College of Computer Science and Electronic Engineering, Hunan University, Changsha, China; ^2^Academy of Military Sciences, Beijing, China; ^3^Center of Rehabilitation Diagnosis and Treatment, Hunan Provincial Rehabilitation Hospital, Changsha, China; ^4^School of Computer Science and Artificial Intelligence, Wuhan Textile University, Wuhan, China

**Keywords:** graph neural network, multi-data heterogeneous networks, microbe-disease association, biological network, graph convolution neural network

## Abstract

The research on microbe association networks is greatly significant for understanding the pathogenic mechanism of microbes and promoting the application of microbes in precision medicine. In this paper, we studied the prediction of microbe-disease associations based on multi-data biological network and graph neural network algorithm. The HMDAD database provided a dataset that included 39 diseases, 292 microbes, and 450 known microbe-disease associations. We proposed a Microbe-Disease Heterogeneous Network according to the microbe similarity network, disease similarity network, and known microbe-disease associations. Furthermore, we integrated the network into the graph convolutional neural network algorithm and developed the GCNN4Micro-Dis model to predict microbe-disease associations. Finally, the performance of the GCNN4Micro-Dis model was evaluated *via* 5-fold cross-validation. We randomly divided all known microbe-disease association data into five groups. The results showed that the average AUC value and standard deviation were 0.8954 ± 0.0030. Our model had good predictive power and can help identify new microbe-disease associations. In addition, we compared GCNN4Micro-Dis with three advanced methods to predict microbe-disease associations, KATZHMDA, BiRWHMDA, and LRLSHMDA. The results showed that our method had better prediction performance than the other three methods. Furthermore, we selected breast cancer as a case study and found the top 12 microbes related to breast cancer from the intestinal flora of patients, which further verified the model’s accuracy.

## Introduction

In microecology, human microbes, especially intestinal microbes, have been found to play a key role in the generation and development of human complex diseases (Baron, 1996). This discovery provided a new perspective for revealing the inherent pathological mechanism of complex diseases. Microbes are responsible for the development of infectious diseases, such as SARS, MERS, and COVID-19 (Singh et al., 2014; Gong et al., 2022). According to the latest real-time statistics from WHO, 618 million confirmed cases and 6.5 million deaths have been reported globally between the outbreak of COVID-19 up until 9 October 2022 (World Health Organization, 2022). Although the composition, morphology, and functions of microbial communities are well understood and thoroughly studied, systematically analyzing the mechanisms by which human microbes initiate and drive diseases is still a major challenge (Karstens et al., 2018). Generally, the interaction between microbes and diseases can be verified to high accuracy using traditional experimental techniques, which can determine whether a certain microbe is directly or indirectly related to diseases. However, this method requires advanced experimental setup, environmental conditioning, and scientific research skill (Teh et al., 2021). Experimentally identifying the relationship between millions of microbes and human diseases takes a lot of time, highly-skilled human labor, and financial resources. This pinch could be obliviated by combining deep learning methods and biological network methods to identify the potential interactions between microbes and diseases on a large scale, allowing us to systemically understand the pathogenic mechanism of complex human diseases and provide a reference for the prevention, diagnosis, and treatment of diseases (Liu et al., 2021).

To address the challenges above, we propose a graph convolutional neural network approach, termed GCNN4Micro-Dis, for microbe-disease prediction. The key motivation is to model associations between diverse biological domains through a graph neural network.

## Related work

In 2016, Ma et al. (2017) established the Human Microbe-Disease Association Database (HMDAD) by collecting published literature and collating 483 pairs of human microbe-disease association information. These highly-accurate data sources have attracted the attention of the bio information field. Researchers have successively proposed microbe-disease prediction models based on different theories, which can be roughly divided into the following three categories: (1) methods based on network algorithms, (2) methods based on dichotomous local features, (3) Machine learning-based methods.

In network algorithm-based methods, the similarity or heterogeneous network is first constructed, then the association probability is calculated based on the network and the specific network algorithm. In 2017, Chen et al., (2018) proposed the first KATZHMDA, which used the known topological information of microbe-disease association network to infer the potential relationship between microbes and diseases by using the social network relationship prediction method. In this model, the problem of predicting potential associations is transformed into the calculation of the similarity between corresponding nodes according to the length and number of paths connecting two nodes in the network. This model not only exhibited excellent predictive power, but also pioneered the field of microbe-disease prediction. Huang et al. (2017) proposed the path-based human microbe-disease association prediction computing model (PBHMDA), which used a special depth-first search algorithm to traverse all the paths communicated between nodes in the heterogenous network, thereby obtaining the prediction score of each pair of microbe-disease association. Shen et al. (2016) used the restart random walk algorithm to score each candidate microbe-disease pair in the microbe network based on Spearman correlation and the disease network based on symptom similarity. The main advantage of these models is their ability to make full use of the network’s topological information. They also involve few parameters, which greatly reduces the difficulty of parameter selection.

The second type of method is based on dichotomous local features. It considers microbes and diseases as local objects and calculates the final prediction by combining their characteristics. Huang et al., (2017) integrated two independent recommendation models and developed NGRHMDA to infer disease-related microbes. NGRHMDA considers diseases that share the same associated microbes or microbes that share the same associated diseases as neighbors. It then considers microbes and diseases as users and items, respectively, and adopts a collaborative filtering recommendation algorithm for local recommendation to make association predictions. Shen et al. (2018) proposed BiRWMP to predict microbe-disease association. The model first builds the microbe-disease associated-network, then it calculates the correlation between microbes and diseases based on the random walk algorithm, using the disease-to-microbe node as the initial starting point. Since the model is a combination of random walks, the local information of microbes, and the random walk of disease information, it can make better predictions than the one-way random walk model. This method improves the local feature bias by considering different perspectives, solving the noise problem caused by the known uneven distribution of associations in the data set to a certain extent and improving the model’s overall predictive power.

The third category is machine learning-based methods. Wang et al. (2017) proposed LRLSHMDA for predicting potential disease-related microbes. Two objective functions were constructed using the Laplacian Regularized Least Squares classification method. An optimal classifier was trained by combining the known topological information of the microbe-disease association network. Potential disease-associated microbes are eventually inferred. Peng et al. developed ABHMDA, which reveals disease-related microbes through a strong classifier consisting of weak classifiers with corresponding weights. ABHMDA assigns different weights to multiple weak classifiers, which proves that the computational method can achieve satisfactory performance in identifying potential associations between microbes and diseases. This work inspired researchers to further explore more novel and effective computational methods to predict the association between microbes and diseases.

## Materials and methods

### Dataset

The dataset used in this study was downloaded from the newly built Human Microbe-Disease Association Database (HMDAD^1^), which collects human microbe-disease association data from 61 published studies. HMDAD contains 450 verified microbe-disease association records between 292 microbes and 39 diseases (Ma et al., 2017; Table 1).

**Table 1:** Data features of verified microbe-disease association.

Number of diseases	Number of microbes	Number of microbe-disease association
39	292	450

## Microbe-disease heterogeneous network

HMDAD allows the download of data on 39 diseases, 292 microbes, and 450 microbes with known association and disease data. This data can be represented as a microbe-disease binary network, which combines all microbe species (*M* = {*m_1_, m_2_, m_3_, …, m_x_*}) and diseases (*D* = {*d_1_, d_2_, d_3_, …, d_y_*}) as A network node. If the microbe *m_j_* is known to be associated with disease *d_i_*, add an edge between node *m_j_* and *d_i_*. Using the adjacency matrix A ∈ R_x*y_, where *x* and *y* represent the database of different kinds of diseases and the number of microbes, an adjacency matrix A may be constructed. If *d_i_* has been proven to be linked with *m_j_*, then *A*_(*i*,*j*)_ = 1, or 0, resulting in an adjacency matrix A with 39 rows and 292 columns containing 1 s and 0 s.

**Figure 1:**
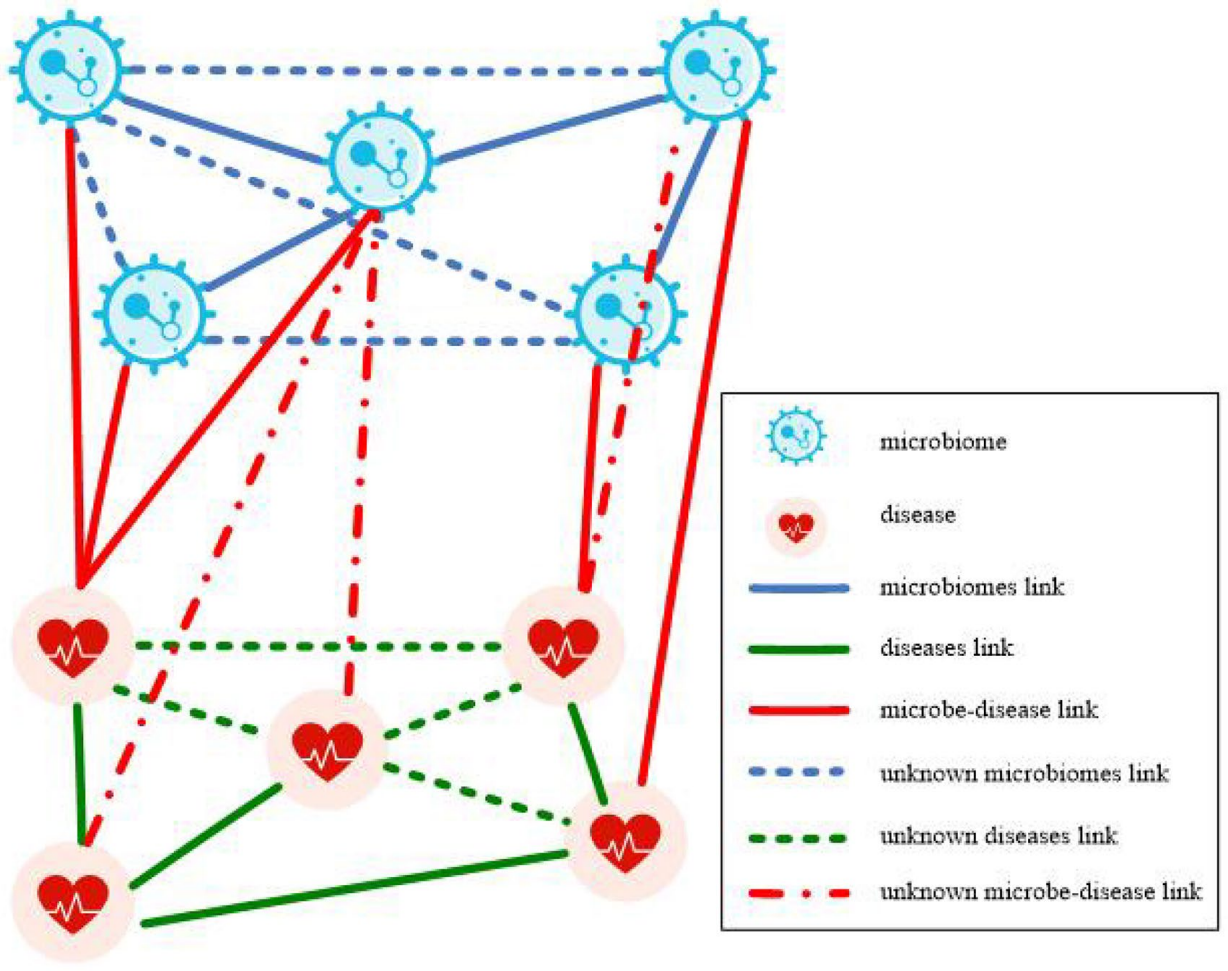
Microbe-disease heterogeneous network.

A microbe-disease heterogeneous network is illustrated in Figure 1. The network is constructed from microbe similarity network, disease similarity network, and known microbe-disease associations. The heterogeneous network contains two node types: microbe nodes and disease nodes, and three types of connecting edges: microbe connecting edges, disease connecting edges, and microbe-disease association edges. The present study aimed to predict the potential association between microbes and diseases using the constructed microbe-disease heterogeneous network, and subsequently find new microbe-disease association pairs with high association possibility from it.

## Graph convolutional neural network

**Figure 2:**
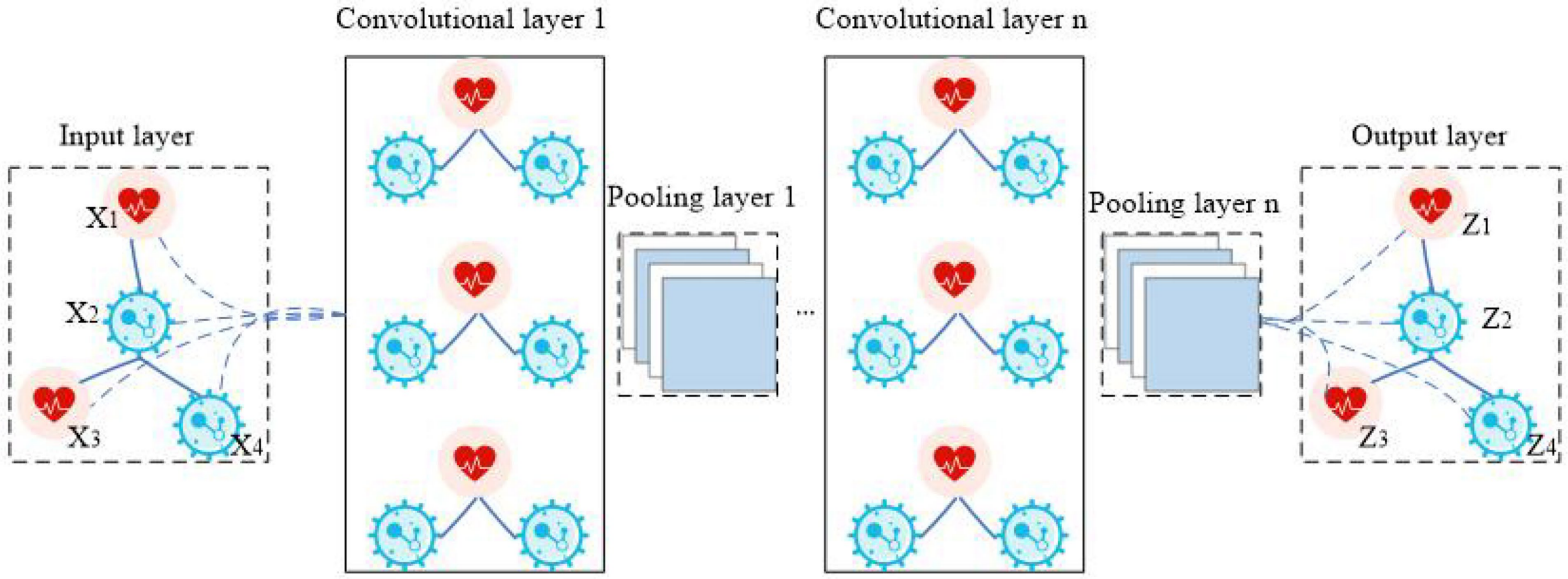
The flowchart of GCNN.

Graph convolutional neural network (GCNN) is a model that applies convolution to the field of graph data (Wu et al., 2021). Its core idea is to learn a mapping function *f*(*x*) by which the characteristics of a node *x* and its neighbors can be aggregated together, resulting in the representation vector of node *x*. In CNN, the image processing method is to further convolve and pool the matrix data by arranging the image pixels into a matrix (LeCun and Bengio, 1995). In GCNN, the image is processed by establishing a topological graph of corresponding relationships between vertices and edges. The spatial features on the topological graph are then extracted (Shou et al., 2022). The structure of GCNN is shown in Figure 2. The biggest difference between GCNN and CNN is that GCNN is stacked at multiple layers, and the parameters between layers are different. The parameters of each layer are shared iteratively. The biggest advantage of GCNN is its introduction of an optimized convolution parameter that extracts graph structure data features. This function is realized through a Laplace matrix in GCNN (Zhang et al., 2022).

GCNNs are divided into two major forms: spatial domain and spectral domain. Spatial domain GCNNs are similar to the application of convolution in deep learning and are optimized to collect information from adjacent nodes. Although this class of network intuitively borrows image convolution operations, it lacks a specific theoretical basis (He et al., 2022). In contrast, spectral domain GCNNs can extract features from nonlinear data more easily. They do so in three steps: (1) perform graphic Fourier transform on input data, (2) convolve the transform result in the spectral domain, (3) inverse Fourier transform convolution result.

Based on graph theory, the coefficient matrix obtained is defined as a graph with nodes and edges. Any graph composed of multiple nodes and edges can be expressed as *G* = (*V*, *E*, *W*), where *V* is a node, *E* is the edge between two nodes, and *W* is the weighted adjacency matrix of connection weights between two vertices. It is usually represented by a Laplace matrix defined as *L* = *D*−*A*, where *D* and *A* represent the degree matrix and adjacency matrix, respectively. The degree matrix is a diagonal matrix representing the number of connected nodes. The adjacency matrix represents the relationship between nodes. Connected nodes are represented as 1, and unconnected nodes are represented as 0. The formula of the Laplace matrix is as follows:


(1) $$L=U\left(\begin{array}{ccc} \lambda_{1} & \; & \;\\ \; & \ddots & \;\\ \; & \; & \lambda_{n} \end{array}\right)U^{-1}={\text{UAU}}^{-1}$$

In Equation 1, *U* is a matrix composed of unit eigenvectors, and *A* is a diagonal matrix composed of the eigenvalues of the Laplace matrix.

## Model performance evaluation metrics

For a prediction model, the model is under-fitted if the deviation is too large, and over-fitted if the variance is too large. A model’s output is strongly distorted when it is under-fitted or over-fitted. To solve these two thorny problems, a set of evaluation methods and performance indicators are needed to comprehensively evaluate the prediction effect of the model. Evaluation methods evaluate the generalizability of the model. Performance indicators evaluate the performance of a single model. The evaluation methods and performance indicators are described in detail below.

Selecting appropriate evaluation methods and performance indicators is important for the evaluation of the model. In this study, common performance index parameters such as accuracy (Acc), recall (Rec), and F1 score (F1) are used (Zhou and Li, 2010). Their definitions are as follows:


(2) $$Acc=\frac{TP+TN}{TP+TN+FN+FP}$$

(3) Rec=TPTP+FN

(4) $$F1=\frac{2^{*}TP}{2^{*}TP+FN+FP}$$

TP represents the number of known microbe-disease association data that can be correctly identified; FP represents the number of unknown microbe-disease association data that have not been correctly identified; TN represents the number of unknown microbe-disease association data that can be correctly identified; FN represents the number of known microbe-disease association data that have not been correctly identified.

The ROC and PR curves were widely used in model evaluation. In the microbe-disease association prediction literature, researchers used the area under the ROC curve (AUC value) and the area under the PR curve (AUPR value) as the comprehensive evaluation indicators of the model. The larger the AUC and AUPR values, the better the predictive power of the model (Zhou and Washio, 2009).

ROC stands for “receiver operating characteristic.” Its vertical axis is the true positive rate (TPR), while its horizontal axis is the false positive rate (FPR). FPR and TPR are calculated using the following formulae:


(5) $$TPR=\frac{TP}{TP+FN}$$

(6) $$FPR=\frac{FP}{FP+TN}$$

TPR represents the proportion of correctly identifying the known microbe-disease associations. FPR represents the proportion of incorrectly identifying the unknown microbe-disease associations. The meanings of TP, FN, FP, and TN have been described in detail in the literature. TP + FN represents all known microbe-disease associations, while FP + TN represents all unknown microbe-disease associations.

PR stands for Precision-Recall. Its vertical axis is Precision (Pre), while its horizontal axis is Recall (Rec). Precision is calculated as follows:


(7) $$\mathrm{Pr}e=\frac{TP}{TP+FP}$$

Precision represents the proportion of correctly predicted known microbe-disease associations in all predicted known microbe-disease associations. Recall represents the proportion of correctly predicted known microbe-disease associations in all known microbe-disease associations.

To sum up, the ROC curve considers both positive and negative samples in the data set: the known microbe-disease associations and the unknown microbe-disease associations. This parameter can be applied to evaluate the overall performance of the model. The PR curve covers only the positive samples, the known microbe-disease associations. It is an indispensable indicator when there is an imbalance between positive and negative samples.

## Results

### Data preprocessing

The positive samples comprise 450 known interactions. The negative samples comprise 450 randomly selected data from the unknown interactions. If the node code of the disease is *d_i_* and the microbe node code is *m_j_*, then the sample code of the interaction between the disease and the microbe is *d_i_ + m_j_*.

### Dataset partition

When evaluating the merits and demerits of a prediction model, the choice of evaluation method is very important. In model evaluation, data sets are commonly divided into training and test sets. The partitioning should satisfy two conditions: the data in the respective sets follow the real distribution, and the data in the sets are mutually exclusive. Considering the different partitioning methods, the evaluation methods are mainly divided into three types: cross-validation, self-help, and set-aside (Zhou and Washio, 2009).

The present study utilized the same assessment method as the existing microbe-disease association predictive models. The proposed model was evaluated using the cross-validation method, specifically 5-fold cross-validation (5-fold CV). For the microbe-disease association data, these three datasets contained only known microbe-disease association data and unknown microbe-disease association data. The known microbe-disease association data were used as positive samples, while the unknown microbe-disease association data were used as negative samples.

Based on the 5-fold CV, all known microbe-disease associations were randomly divided into five groups.

1. Divide the positive samples into five subsets of equal size.
2. Divide the negative samples into five subsets of equal size.
3. One of the five subsets of positive and negative samples takes turns as the test set.
4. Remove the positive samples in the test set from the adjacency matrix by deleting their links with known interactions in the test set network.
5. In the remaining four subsets of positive and negative samples, the training set is 0.875, and the validation set is 0.125.
6. Randomly generate the initialization code of each node.
7. Repeat all experiments five times, with iteration set to 5, and average the final results to reduce the bias caused by random grouping.

### Hyper-parameters selection

Convolutional neural network training can be regarded as a process of minimizing the loss function. The training network must initialize the parameters, set the appropriate learning rate, select the appropriate batch normalization method, and continuously iterate and update the parameters according to the optimization algorithm and strategy, including hyper parameters like Epoch, Batch, Batch_size, iteration, learning rate, etc.

In this experiment, we set Epoch to 100, learning rate to 0.001, coding dimension to 256, and the number of GCN coding layers to 3. Epoch refers to the complete training of the model using all the data in the training set, called “generation training.” Iteration is the process of updating the model parameters using a Batch of data, called “a training session.” The learning rate determines how fast the parameters move to the optimal value. If the learning rate is too large, it is likely to cross the optimal value and lead to function convergence failure or even divergence. On the contrary, if the learning rate is too low, the optimization becomes inefficient, the convergence is too slow, and the algorithm can easily fall into a local optimum. The appropriate learning rate should converge as soon as possible on the premise of ensuring convergence.

### Model effects

Samples with the same number of positive samples were randomly selected as negative samples from the unknown samples to ensure the balance of positive and negative samples. The 5-fold CV method was used to ensure that each sample data was used as a test set. The experiment was repeated five times, which greatly reduced the influence of randomness. The 25 experimental results reported 19 AUC values that are mostly above 0.8 with an average value of 0.8154, indicating that the model can be well applied to predict the link between diseases and microbes.

There is still a lot of room to improve the model’s performance. Its results are largely limited by the amount of data, with only 450 positive samples utilized in this study. Furthermore, the node initialization coding adopted random initialization coding, which cannot express the inherent attribute characteristics of different node entities well.

The average AUC value and standard deviation given by the model was 0.8954 ± 0.0030. Our model evidently performed well and can help identify novel disease-microbe associations (Table 2).

**Table 2:** The summary of model performance under 5-fold CV.

		Iter1	Iter2	Iter3	Iter4	Iter5
Fold0	Acc	0.7556	0.7722	0.7722	0.7722	0.7833
	Rec	0.7444	0.7556	0.7444	0.7333	0.7778
	F1	0.7528	0.7684	0.7657	0.7630	0.7821
	AUC	0.8121	0.8169	0.8223	0.8254	0.8328
	AUPR	0.7866	0.8071	0.8148	0.8223	0.8065
Fold1	Acc	0.7444	0.7333	0.7333	0.7556	0.7722
	Rec	0.7444	0.7667	0.7889	0.8111	0.7778
	F1	0.7444	0.7419	0.7474	0.7684	0.7735
	AUC	0.8020	0.8137	0.8230	0.8181	0.8207
	AUPR	0.7661	0.8146	0.8138	0.7945	0.7913
Fold2	Acc	0.7444	0.7222	0.7444	0.7278	0.7556
	Rec	0.7333	0.7556	0.7556	0.7667	0.7556
	F1	0.7416	0.7312	0.7473	0.7380	0.7556
	AUC	0.8258	0.8084	0.8226	0.7947	0.8126
	AUPR	0.8279	0.8282	0.8283	0.7794	0.8125
Fold3	Acc	0.7389	0.6833	0.7278	0.7333	0.7278
	Rec	0.7444	0.6667	0.7444	0.7222	0.7222
	F1	0.7403	0.6780	0.7322	0.7303	0.7263
	AUC	0.7795	0.7670	0.7985	0.7968	0.7974
	AUPR	0.7906	0.7539	0.7866	0.7919	0.7713
Fold4	Acc	0.7722	0.7556	0.7611	0.7556	0.7611
	Rec	0.7333	0.7111	0.7333	0.7000	0.6889
	F1	0.7630	0.7442	0.7543	0.7412	0.7425
	AUC	0.8485	0.8204	0.8338	0.8260	0.8190
	AUPR	0.8468	0.8250	0.8164	0.8237	0.7981

**Figure 3:**
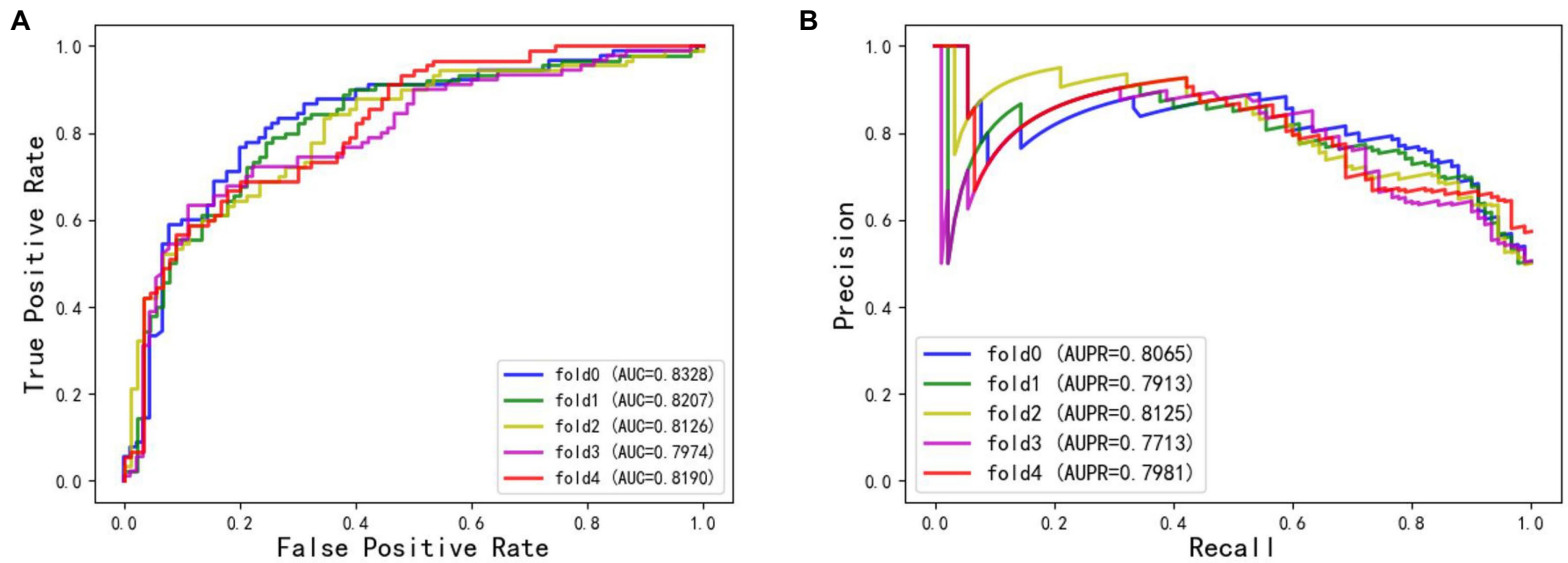
**(A)** The ROC curves of Iter5. **(B)** The AUPR curves of Iter5.

The ROC and AUPR curves of the fifth experiment (Iter5) are shown in Figure 3.

## Comparison with other methods

To verify the superiority of the GCNN4Micro-Dis model proposed in this study, it is compared with three advanced methods used to predict microbe-disease associations: KATZHMDA (Chen et al., 2018), BiRWHMDA (Zou et al., 2017), and LRLSHMDA (Wang et al., 2017).

- The KATZ measure for Human Microbe-Disease Association (KATZHMDA) is a novel computational model based on the assumption that functionally similar microbes tend to have similar interaction and non-interaction patterns with non-infectious diseases and vice versa (Chen et al., 2018).
- BiRWHMDA is a novel computational model to predict potential microbe-disease associations using bi-random walk on the heterogeneous network (Zou et al., 2017).
- The Laplacian Regularized Least Squares for Human-Microbe Disease Association (LRLSHMDA) is a semi-supervised computational model using the Gaussian interaction profile kernel similarity calculation and Laplacian regularized least squares classifier (Wang et al., 2017).

The AUC of BiRWHMDA reached 0.7984, while the AUCs of LRLSHMDA and KATZHMDA were 0.8410 and 0.8428, respectively. The AUC of GCNN4Micro-Dis was better than that of BiRWHMDA. Therefore, the performance of GCNN4Micro-Dis was not different from the other three methods in terms of prediction accuracy.

The data set used in this study was unbalanced, making the AUPR value an indispensable model evaluation index. The AUPR of LRLSHMDA, KATZHMDA, and BiRWHMDA were 0.5045, 0.4782, and 0.4363, respectively. The AUPR of GCNN4Micro-Dis was 0.8092, better than the other three competitors. The experimental data conclusively demonstrated that GCNN4Micro-Dis had a better prediction performance than the other three methods (Table 3).

**Table 3:** Comparison of AUC and AUPR for different microbe-disease association predictions methods.

Methods	AUC	AUPR
GCNN4Micro-Dis	**0.8154**	**0.8092**
LRLSHMDA (Wang et al., 2017)	0.8410	0.5045
KATZHMDA (Chen et al., 2018)	0.8428	0.4782
BiRWHMDA (Zou et al., 2017)	0.7984	0.4363

Bold values represent the effect of our model.

## Case study

In this section, a prevalent human disease, breast cancer, was selected as a case study to further analyze the performance of GCNN4Micro-Dis. Given that the role of gut microbiome in health and disease has recently attracted more and more attention, many observations and *in vitro* studies depict that it may be involved in the development of breast cancer. The 12 microbes most related to breast cancer were selected from the intestinal flora of patients as case studies. The result has been verified in the literature (Liu et al., 2020; Huang et al., 2021). Some fecal intestinal bacteria were found to be associated with breast cancer and are expected to become new targets for breast cancer treatment (Wu et al., 2016; Zheng et al., 2018; Table 4).

**Table 4:** Top 12 potential microbes related to breast cancer.

BRCA subtypes	Rank	Microbes
HER2 positive	1	Megasphaera
	2	Barnesiellaceae
	3	Alloprevotella
ER positive	1	Megasphaera
	2	Roseburia
	3	Prevotellaceae
PR positive	1	Prevotellaceae
	2	Tyzzerella
	3	Enorma
Ki67 positive	1	Tenericutes
	2	Izimaplasmatales
	3	Sporobacter

## Conclusion

A heterogeneous network of microbe-disease association was constructed from data extracted from the HMDAD database. A graph neural network algorithm was proposed, and the accuracy of our algorithm was evaluated using a 5-fold cross-validation. The main parameters involved in the algorithm were verified, proving the effectiveness of the prediction method. The main research results of this paper are as follows.

GCNN4Micro-Dis, a microbe-disease prediction method based on the Graph Neural Network and Multi-Data Heterogeneous Networks, was proposed. The heterogeneous network was obtained by integrating the known microbe-disease networks. The network was applied to the Graph Neural Network model for prediction. The methods proposed in this study predicted the association between potential microbes and diseases. Although these methods performed well in experimental verification and analysis, there are still some limitations that could be addressed in future works:

(1) The known microbe-disease association dataset was too small, which reduced its accuracy to some extent. In the future, the method’s predictive power will improve with more data available. (2) More similarity data can be added. The microbe and disease similarity in this paper are calculated from the known microbe-disease associations, which were inadequate. The prediction could be more accurate if more similarity data could be integrated into the heterogeneous networks. (3) More network information can be added. The current prediction methods require known microbe disease association data. Without this information, most methods cannot be implemented. More information may be mined if the potential microbe disease association can be predicted without this information. For example, the correlation data between microbes and RNA and between RNA and microbes allows the use an RNA network as an intermediate layer to build a three-layer microbe RNA disease network. The three-layer heterogeneous network can mine more unknown information.

Due to the relatively late development of microbe-disease association prediction, there are still many deficiencies and challenges at the present stage. Nevertheless, many studies have made preliminary exploration on the design of the prediction model (Peng et al., 2017, 2021, 2022a,b; Shen et al., 2022), which can be summarized as follows:

1. There are relatively few validated microbe-disease association data. Relatively few microbe-disease associations have been demonstrated through biological experiments compared to other biomarkers, such as non-coding RNAs. Since current computational methods often infer possible microbe-disease associations based on known association data, more known associations are needed to enrich the training set of the prediction models and improve their prediction power. Therefore, more accurate microbe-disease associations should be mined, using biological experiments as the fundamental data source for the calculation methods.
2. Few available datasets. The number of publicly available microbe-disease association databases is limited, yet few researchers have constructed new data sets, forcing a broad consensus of data sets used in the field. Most of the data sets used currently are microbe-disease associations provided by the HMDAD database. Although they are true and reliable associations verified by biological experiments, the number is small. Small and single data sets cannot fully depict the performance of the prediction model and render the prediction model unreliable. Therefore, there is an urgent need to build a larger microbe-disease association database.
3. The design of some methods should be improved. Methods based on network algorithms usually make assumptions about probability distributions, which fail if the data sources are not conformant. For example, this part of the model constructs similarity networks by assuming that functionally similar microbes have similar interaction patterns with diseases, which is more beneficial for microbes with more known related diseases. Optimizing the network structure by introducing local features is expected to improve this deficiency.
4. The prediction performance must be improved. Microbe-disease association prediction is a relatively new research field, so the performance of the proposed prediction models must be improved. In the future, more diverse biological information and more effective computational methods (such as neural networks) can be used to design prediction models with superior performances.

As an unsupervised deep neural network, GCN can learn and extract features from unlabeled data, obtain low-dimensional feature expressions from high-dimensional original data, simplify the classification work, and overcome the randomness of weight coefficient initialization in traditional neural networks. In future works, biological information features, such as functional similarity of microbes and semantic similarity of diseases, will be considered for addition to GCNN4Micro-Dis to more accurately predict the associations between microbes and diseases and help prevent, diagnose, treat, and prognose diseases.

## Data availability statement

The original contributions presented in the study are included in the article/supplementary material, further inquiries can be directed to the corresponding authors.

## Author contributions

HG and MJ: conceptualization. JX and XY: methodology. YM and HZ: software. HZ and SY: validation. XY: resources, supervision, and funding acquisition. SY: data curation. HG: visualization and writing-original draft preparation. HG and JX: writing-review and editing. MJ and JX: project administration. All authors have read and agreed to the published version of the manuscript.

## Funding

This work was supported by the Natural Sciences Foundation of Hunan Province (Grant No. 2021JJ30139), the National Natural Science Foundation of China (Grant No. 61773157), the Key Project of R & D plan of Changsha (Grant No. kq2004011), the China Postdoctoral Science Foundation (Grant No. 2022M711113), the Rehabilitation Project of Hunan Disabled Persons’ Federation in 2022 (Grant No. 2022XK0305), and the Excellent Youth Fund of Hunan Provincial Department of Education (Grant No. 22B0021).

## Conflict of interest

The authors declare that the research was conducted in the absence of any commercial or financial relationships that could be construed as a potential conflict of interest.

## Publisher’s note

All claims expressed in this article are solely those of the authors and do not necessarily represent those of their affiliated organizations, or those of the publisher, the editors and the reviewers. Any product that may be evaluated in this article, or claim that may be made by its manufacturer, is not guaranteed or endorsed by the publisher.

## Footnotes

^1^ http://www.cuilab.cn/hmdad
